# Effect of four rounds of annual school‐wide mass praziquantel treatment for *schistosoma mansoni* control on schistosome‐specific immune responses

**DOI:** 10.1111/pim.12530

**Published:** 2018-04-29

**Authors:** E. M. Ndombi, B. Abudho, N. Kittur, J. M. Carter, H. Korir, D. K. Riner, H. Ochanda, Y‐M. Lee, W. E. Secor, D. M. Karanja, D. G. Colley

**Affiliations:** ^1^ Kenya Medical Research Institute Centre for Global Health Research Kisumu Kenya; ^2^ School of Biological Sciences University of Nairobi Nairobi Kenya; ^3^ Department of Pathology Kenyatta University Nairobi Kenya; ^4^ Department of Biomedical Sciences Maseno University Maseno Kenya; ^5^ Center for Tropical and Emerging Global Diseases University of Georgia Athens GA USA; ^6^ Division of Parasitic Diseases and Malaria Centers for Disease Control and Prevention Atlanta GA USA; ^7^ Department of Microbiology University of Georgia Athens GA USA

**Keywords:** antibodies, cytokines, mass drug administration, praziquantel, schistosomiasis, school‐based treatment

## Abstract

This study evaluated potential changes in antischistosome immune responses in children from schools that received 4 rounds of annual mass drug administration (MDA) of praziquantel (PZQ). In a repeated cross‐sectional study design, 210 schistosome egg‐positive children were recruited at baseline from schools in western Kenya (baseline group). Another 251 children of the same age range were recruited from the same schools and diagnosed with schistosome infection by microscopy (post‐MDA group). In‐vitro schistosome‐specific cytokines and plasma antibody levels were measured by ELISA and compared between the 2 groups of children. Schistosome soluble egg antigen (SEA) and soluble worm antigen preparation (SWAP) stimulated higher IL‐5 production by egg‐negative children in the post‐MDA group compared to the baseline group. Similarly, anti‐SEA IgE levels were higher in egg‐negative children in the post‐MDA group compared to the baseline group. Anti‐SEA and anti‐SWAP IgG4 levels were lower in egg‐negative children in the post‐MDA group compared to baseline. This resulted in higher anti‐SEA IgE/IgG4 ratios for children in the post‐MDA group compared to baseline. These post‐MDA immunological changes are compatible with the current paradigm that treatment shifts immune responses to higher antischistosome IgE:IgG4 ratios in parallel with a potential increase in resistance to reinfection.

## 1 INTRODUCTION

Epidemiological studies of human schistosome infections in endemic areas demonstrate an age‐dependent infection pattern and the possibility of a slow development of protective immunity. Antigens released from dying worms are hypothesized to provide the stimulus for the development of protective immune responses over time.^1^ The induction of a protective response is thought to require exposure to a certain threshold level of the responsible antigen(s)^2^ and may be accelerated by repeated treatments with PZQ following reinfections, as treatment may lead to the release of previously hidden parasite antigens.^3^, ^4^, ^5^ While this increased resistance to reinfection seldom leads to sterile immunity,^6^ it could assist in maintaining lower levels of infection.

Several immunologic correlates of protection against reinfection by schistosomes have been reported and are mainly Th2‐associated.^7^, ^8^, ^9^ These include elevated parasite‐specific IgG1 and IgE and antitegumental‐allergen‐like 1 (TAL‐1) IgE, higher IgE:IgG4 ratios to schistosome egg or worm antigens and increased number of eosinophils.^7^, ^8^, ^10^, ^11^ Elevated IL‐4 and IL‐5 production and levels following treatment are also associated with resistance to reinfection, while experimental mouse studies have shown that IL‐10 can block the development of post‐treatment protective responses.^8^, ^9^, ^12^

Annual mass drug administration (MDA) of PZQ to school children in schistosomiasis endemic areas is an integral component of the school‐based deworming programme in many countries. In 2012, MDA with PZQ was included for the first time in a Kenyan nationwide school‐based deworming (SBD) campaign that also included albendazole for soil‐transmitted helminths (STH).^13^ So far, 5 rounds of annual SBD have been administered since the launch of the programme, with a target of reaching at least 75% of school‐age children as recommended by the WHO.^14^ While the main intended benefits of MDA are to reduce morbidity and improve a child's developmental potential, the concomitant effects of these treatments on a child's antischistosome immune responses are largely unknown. Also, unknown are the consequences of these potentially altered immune responses in regard to potential future reinfections or other health‐related outcomes. Thus, millions of children are now undergoing annual MDA with PZQ without data regarding its impact on their immune responses to schistosome antigens. Therefore, we have studied the effects of consecutive 4 years of annual school‐based MDA on antischistosome immune responses previously associated with immunoregulation and resistance to reinfection.

## 2 MATERIALS AND METHODS

This repeated cross‐sectional study was conducted as among school children in the Asembo area of Rarieda Sub‐county in Siaya County of western Kenya. The design was to evaluate the antischistosome immunologic impact of multiple annual school‐based MDAs in the entire student population attending the 5 study schools. Treatments during the MDAs were provided by the study team and administered as directly observed therapy (DOT). School attendance in these schools, based on assessments by teachers was >95%. In addition, due to widespread publicity and the provision of porridge and juice, attendance on MDA days surpassed that and often included nonschool attending siblings. The study protocol was reviewed by the Scientific and Ethical Review Unit (SERU) of the Kenya Medical Research Institute (KEMRI) as well as institutional review boards of the University of Georgia and the Centers for Disease Control and Prevention (CDC) and commenced only after necessary approvals had been granted. Parents or guardians of participating children gave informed consent, and children gave assent prior to being enrolled in the study. A total of 210 schistosomiasis egg‐positive participants of ages 10‐19 year old were recruited from 2 primary and 3 secondary schools in western Kenya, all within 5 km radius of Lake Victoria at baseline in 2012. After 5 years of their schools having received 4 rounds of annual MDA with PZQ and albendazole, 251 participants from across the same age spectrum were recruited from the same schools. Participants were at least in the fifth grade at baseline, and in the post‐MDA group, and would therefore have had an opportunity to receive PZQ at least 4 times. The proportion of children drawn from each grade in each of the 5 schools remained the same at both time points, as did the age and sex structure. Prior to baseline and the fifth year's annual MDA, stool samples were collected from each participant on 3 consecutive days and examined by the Kato‐Katz faecal microscopy method for diagnosis of schistosome and STH infections,^15^ using 2 slides per stool. Infection intensity was expressed in eggs per gram of faeces (epg), and arithmetic means were calculated. About 10 mL of venous blood was collected by venipuncture by a trained phlebotomist into heparin vacutainer tubes from each participant and transported to the immunology laboratory at the Centre for Global Health Research (CGHR) of KEMRI, in Kisian, near Kisumu city, Kenya, for storage and analysis. A portion of the blood was used to prepare blood smears for malaria diagnosis. The rest of the blood was used for whole‐blood cultures for cytokine production or centrifuged to obtain plasma for antibody measurements.

### 2.1 laboratory procedures

#### 2.1.1 Cytokine production and evaluation

1.75 mL of the heparinized blood was used to set up whole‐blood cultures for evaluation of in vitro production of cytokines (IL‐5, IL‐10, IL‐13, IFN‐γ) as previously described.^16^ Briefly, blood was diluted 1:5 in culture media (RPMI 1640, 1% L‐glutamine, 1% Penicillin‐streptomycin). 1.5 mL per well of this mixture was used for whole‐blood cultures in Corning^®^ Costar^®^ 24‐well tissue culture plates (Corning Inc., New York), with either no added stimulant or one of the following potential stimulants: phytohemagglutinin‐P (PHA; Sigma‐Aldrich, St. Louis, MO) at a final concentration of 2.5 μg/mL, schistosome soluble worm antigen preparation (SWAP) 10 μg/mL final concentration, soluble egg antigen (SEA) at final concentration of 5 μg/mL.^17^ The cultures were maintained for either 3 days (PHA and controls) or 5 days (antigens and controls) in a 5% CO_2_ incubator at 37°C. Culture supernatant fluids were then harvested into cryovials and stored frozen (−20°C) until assayed together later for cytokine levels. Cytokine levels were measured using Duoset ELISA kits (R&D systems, Minneapolis, MN) as per manufacturer's protocol, with modifications as follows: (i) the detection antibody was left to incubate in the plate for 1 hour and (ii) an 8‐point standard curve was used instead of 7, with the lowest standard being 0 pg/mL.

#### 2.1.2 Serologic assays for schistosome‐specific antibodies of given isotypes

Heparinized whole blood (~5.75 mL) was centrifuged to yield approximately 3 mL of plasma, which was stored in aliquots frozen at −20°C. Assays were performed to detect antischistosome SEA or SWAP‐specific total IgG, IgG4 and IgE or TAL‐1‐, TAL‐2‐ and TAL‐5‐specific IgE and IgG4 as previously described^18^, ^19^, ^20^ with modifications described below. Recombinant TAL antigens were produced at the CDC from plasmids containing the TAL genes kindly provided by Colin Fitzsimmons, Cambridge University.^21^ Final concentrations of coating antigens were prepared as follows: 5 μg/mL for SWAP, 2.5 μg/mL of SEA for IgE, 0.625 μg/mL of SEA for IgG and IgG4, 1.5 μg/mL of TAL 1, 4.0 μg/mL of TAL 2 and 8.0 μg/mL of TAL 5. Samples were added to the plate in duplicate at a dilution of 1:20 for IgE measurement and 1:50 for IgG or IgG4 measurement. A pool made from plasmas of 12 Kenyan car washers known to be high responders to SWAP, and therefore likely to have responsiveness to TAL antigens was included on each plate. The highest standard was assigned 1000 arbitrary units, and twofold serial dilutions were used to make an 8‐point standard curve. The lowest standard, containing buffer alone, was assigned 0 units. Normal human serum (NHS) obtained from 20 volunteers who resided in North America and had never been infected with human or bird schistosomes was used for negative controls. Horseradish Peroxidase (HRP)‐conjugated isotype‐specific secondary antibody (eBiosciences, Thermo Fisher Scientific, Massachusetts) was diluted in buffer at the ratio of 1:1000 for IgE and 1:50 000 for IgG/IgG4. A final concentration for each sample was obtained by subtracting the average NHS value on a plate from the average concentration of each sample run on the same plate.

### 2.2 Data processing and analysis

Data were analysed using GraphPad Prism software version 6 (GraphPad Inc., California, USA). Both baseline data and final year measures were non‐normally distributed; hence, analyses were mainly performed using nonparametric tests. Mann‐Whitney *U* or Kruskal‐Wallis with Dunns's multiple comparison post‐test was used to compare medians for 2‐sample and multiple‐sample comparisons, respectively.

## 3 RESULTS

### 3.1 Demographic characteristics of study participants

A total of 210 *Schistosoma mansoni‐*infected (egg positive) school children were recruited at baseline from 2 primary and 3 secondary schools from within 5 km of Lake Victoria. These schools subsequently received 4 years of annual, school‐based MDA, and all students present at the times of the annual MDAs were treated, with annual coverage approaching 95%. Treatment with PZQ (40 mg/kg body weight) and albendazole (400 mg) was directly observed by the study team.

Participant ages ranged from 10 years to 19 years with 15 as the median age at both baseline and after 4 years of annual MDA. At baseline, 102 (48.6%) of the participants were boys; in the post‐MDA group, 131 (52.2%) were boys (Table 1). At baseline, 122 participants (58.1%) had light infections (1‐100 epg), while 70 (33.3%) had moderate infections (101‐399 epg) and 18 (8.6%) had heavy infections (≥400 epg), based on WHO recommended categorization of *S. mansoni* infection intensity.^22^, ^23^ The children in this immunologic study were a subset of the entire enrolment of all 5 schools that participated in a broader MDA study.^24^ The infection intensities for all screened children pertinent to this study (Grade 5 onward) were at baseline as follows: 53.9%, 33.3% and 12.7%, light, moderate and heavy, respectively, indicating that our random selection among *S. mansoni*‐positive only children did not select for only heavily infected children. In the post‐MDA group, 205 (81.7%) of the participants were egg‐negative, and of the 18.3% who were egg‐positive; none had heavy infections, while 2 (0.8%) and 44 (17.5%) had moderate infections and light infections, respectively. Prevalence of STH and malaria was 20.5% and 29.8%, respectively, at baseline and 1.6% and 30.7%, respectively, in the post‐MDA group. Upon analysis, there were no differences in any of the antischistosome responses based on the presence or absence of positive malaria smears or STH results. In addition, examining any of the immunologic data in relationship to the epg values of *S. mansoni*‐positive children did not yield any statistical correlations.

**Table 1:** Demographic characteristics of study participants at baseline and after 4 years of MDA (post‐MDA group)

Characteristic		Baseline (n = 210)	Post‐MDA Group (n = 251)
Male sex, n (%)		102 (48.6)	131 (52.2)
Age (years), median		15	15
*Schistosoma mansoni* egg count: arithmetic mean (95% CI) of *S. mansoni*‐positive individuals, epg		160 (126‐ 193)	29 (11‐ 46)
Proportion with *S. mansoni* infection intensity, n (%)	Negative (0 epg)	0 (0)	205 (81.7)
	Light (1‐99 epg)	122 (58.1)	44 (17.5)
	Moderate (100‐399 epg)	70 (33.3)	2 (0.8)
	Heavy (≥400 epg)	18 (8.6)	0 (0)
STH prevalence, n (%)		43 (20.5)	4 (1.6)
Malaria prevalence, n (%)		62 (29.8)	77 (30.7)

CI, confidence interval; epg, eggs per gram; STH, soil‐transmitted helminths; MDA, mass drug administration.

### 3.2 Cytokine production

Cytokine levels are presented as each cytokine (IL‐5, IL‐10, IL‐13, IFN‐γ) stimulated by either PHA (Day 3 harvest) or SEA or SWAP (Day 5 harvest) and are reported as Experimental minus Media Control values (E‐C). The mean media control values for IL‐5, IL‐13 and IFN‐g did not differ between baseline values and those after the 4 MDAs. The mean media control value for IL‐10, however, did differ and is described below in the section on IL‐10.

#### 3.2.1 IL‐5

**Figure 1:**
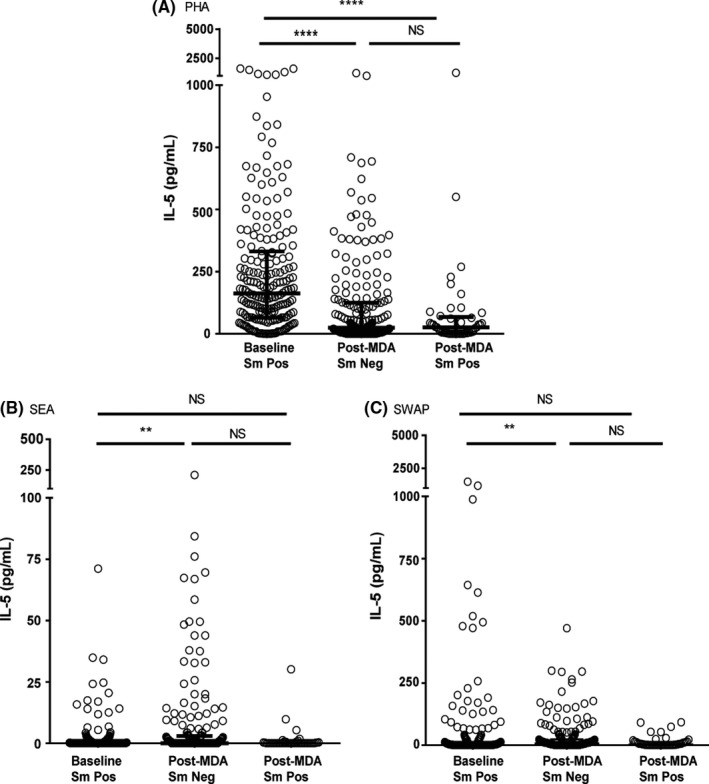
IL‐5 levels following stimulation with PHA (A, 3 day cultures), soluble egg antigen (SEA) (B) and soluble worm antigen preparation (SWAP) (C) (5 day cultures) at baseline for children who were all *Schistosoma mansoni* egg‐positive, and after 4 mass drug administrations (MDAs) (post‐MDA group) in children who were either *S. mansoni* egg‐negative or egg‐positive. Cytokine levels are less control values. Data are presented as dot plots with medians at centreline and whiskers at 25th and 75th percentile. Comparison was done by Kruskal‐Wallis followed by Dunn's multiple comparison test. **P < .01 and **^**^ *P* < .0001

Figure 1 shows IL‐5 levels at baseline and in egg‐negative and egg‐positive children in the post‐MDA group. Significantly more IL‐5 was produced following PHA stimulation in the baseline group than the post‐MDA group, regardless of a child's infection status at the end of the study. This is unlike IL‐5 production in response to SEA or SWAP stimulation, where the levels of the cytokine produced in response to the schistosome antigens were higher in the egg‐negative children in the post‐MDA group than the baseline group but were not higher among the children who were egg‐positive at the end of the study. It should be noted, however, that baseline IL‐5 responses to SWAP appear higher than responses in the post‐MDA *S. mansoni*‐negative children as shown in Figure 1. This is due to greater variability and very high responses by a few individuals in the baseline group, but a lower median response compared to the *S. mansoni*‐negative post‐treatment group.

#### 3.2.2 IL‐10

**Figure 2:**
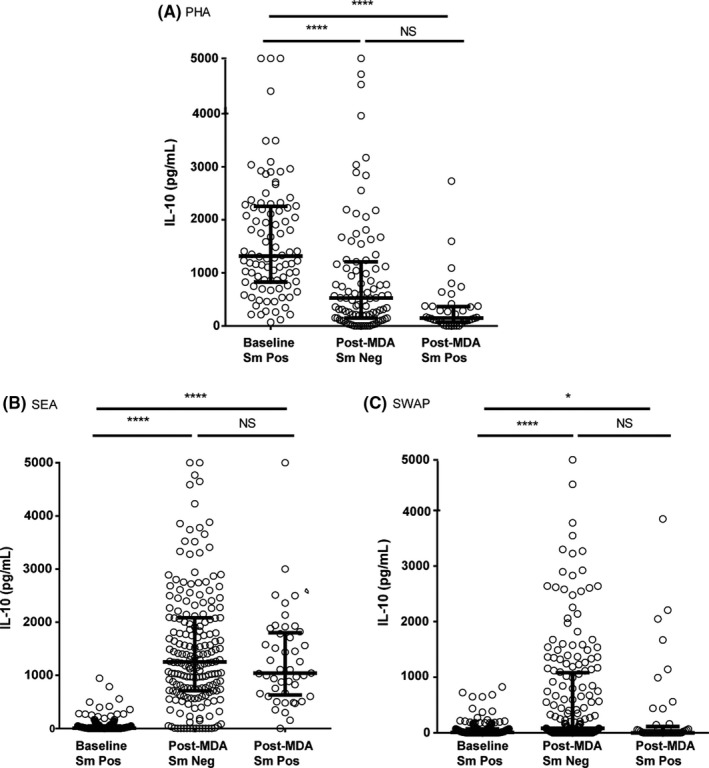
IL‐10 levels following stimulation with PHA (A, 3 day cultures), soluble egg antigen (SEA) (B) and soluble worm antigen preparation (SWAP) (C) (5 day cultures) at baseline for children who were all *Schistosoma mansoni* egg‐positive, and after 4 mass drug administrations (MDAs) (post‐MDA group) in children who were either *S. mansoni* egg‐negative or egg‐positive. Cytokine levels are less control values. Data are presented as dot plots, medians at centreline and whiskers at 25th and 75th percentile. Comparison was done by Kruskal‐Wallis followed by Dunn's multiple comparison test. *P < .05, **^**^ *P* < .0001

Both 3 day and 5‐day media control cultures yielded high levels of IL‐10, without exogenous stimulation. While observed in both the baseline and post‐MDA groups, this spontaneous production of IL‐10 was much lower in the post‐MDA group (*P* < .0001). Consequently, IL‐10 data are shown both as stimulant minus control (Figure 2) and as stimulant vs control media values (Figure S1). Levels of IL‐10 produced in response to PHA were significantly higher at baseline compared to levels after 4 MDAs, regardless of infection status. Stimulation of IL‐10 production by SEA was, by contrast, significantly higher in the post‐MDA group than at baseline, whether the child was infected or not. A similar pattern was observed upon exposure to SWAP, except that overall, SWAP stimulation led to the production of much lower levels of IL‐10 than SEA stimulation in both the baseline and post‐MDA groups.

#### 3.2.3 IL‐13

**Figure 3:**
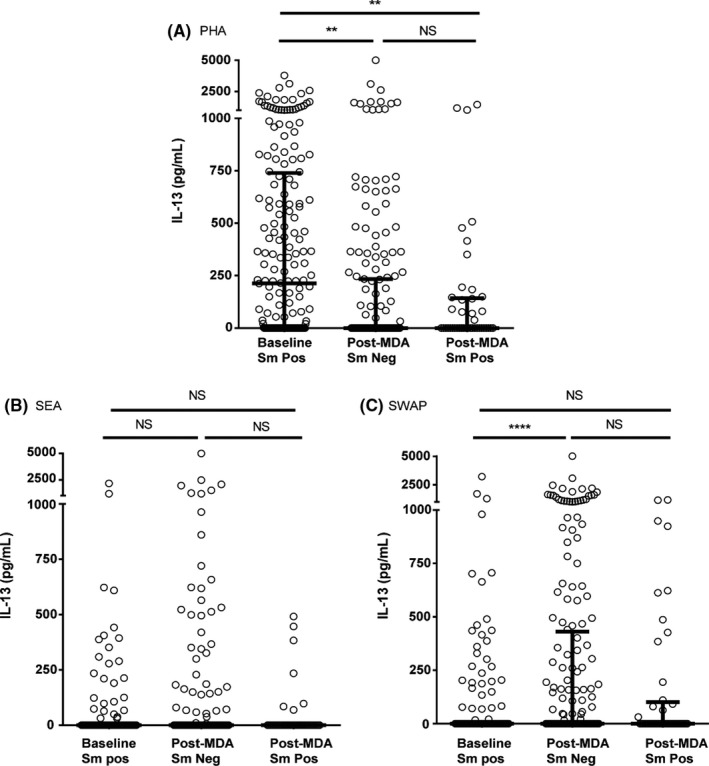
IL‐13 levels following stimulation with PHA (A, 3 day cultures), soluble egg antigen (SEA) (B) and soluble worm antigen preparation (SWAP) (C) (5 day cultures) at baseline for children who were all *Schistosoma mansoni* egg‐positive, and after 4 mass drug administrations (MDAs) (post‐MDA group) in children who were either *S. mansoni* egg‐negative or egg‐positive. Cytokine levels are less control values. Data are presented as dot plots, medians at centreline and whiskers at 25th and 75th percentile. Comparison was done by Kruskal‐Wallis followed by Dunn's multiple comparison test. *P < .05, **^**^ *P* < .0001

Figure 3 depicts IL‐13 levels following stimulation with PHA, SEA and SWAP in whole‐blood cultures from children in the baseline and post‐MDA groups. In the baseline group, PHA stimulated a higher level of IL‐13 production. In the post‐MDA group, PHA stimulated less IL‐13 production, regardless of whether a child was infected or not. There were no significant differences between groups in IL‐13 production in response to SEA. SWAP stimulation of IL‐13 production was higher among egg‐negative children in the post‐MDA group than the baseline group.

#### 3.2.4 IFN‐γ

**Figure 4:**
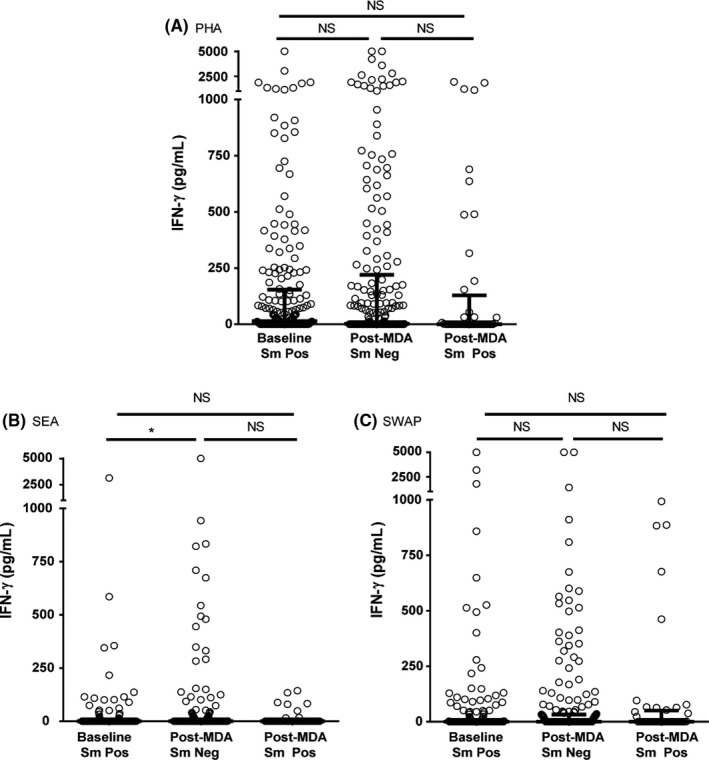
IFN‐γ levels following stimulation with PHA (A, 3 day cultures), soluble egg antigen (SEA) (B) and soluble worm antigen preparation (SWAP) (C) (5 day cultures) at baseline for children who were all *Schistosoma mansoni* egg‐positive, and after 4 mass drug administrations (MDAs) (post‐MDA group) in children who were either *S. mansoni* egg‐negative or egg‐positive. Cytokine levels are less control values. Data are presented as dot plots, medians at centreline and whiskers at 25th and 75th percentile. Comparison was done by Kruskal‐Wallis followed by Dunn's multiple comparison test. *P < .05

In the post‐MDA group, children who were egg‐negative produced more IFN‐γ in response to SEA, than children at baseline (Figure 4). There was no significant difference in IFN‐γ production by PHA or SWAP stimulation between children at baseline and those in the post‐MDA group.

### 3.3 Anti‐SEA, anti‐SWAP and anti‐TAL antibodies

**Figure 5:**
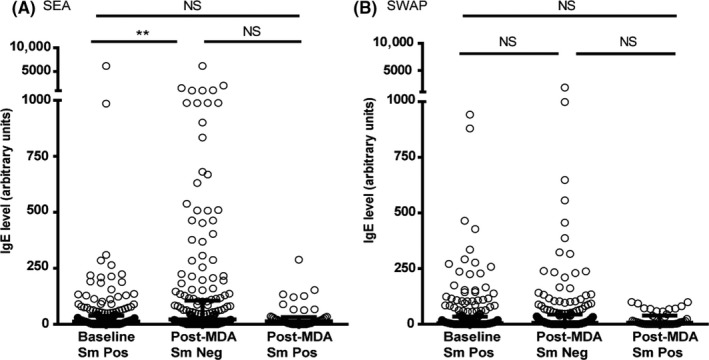
Anti‐(SEA) soluble egg antigen IgE levels (A) and anti‐ soluble worm antigen preparation (SWAP) IgE levels (B) at baseline for children who were all *Schistosoma mansoni* egg‐positive, and after 4 mass drug administrations (MDAs) (post‐MDA group) in children who were egg‐negative and those who were egg‐positive. Data are presented as dot plots, medians at centreline, and whiskers at 25th and 75th percentile. Comparison was done by Kruskal‐Wallis followed by Dunn's multiple comparison test. **P < .01

**Figure 6:**
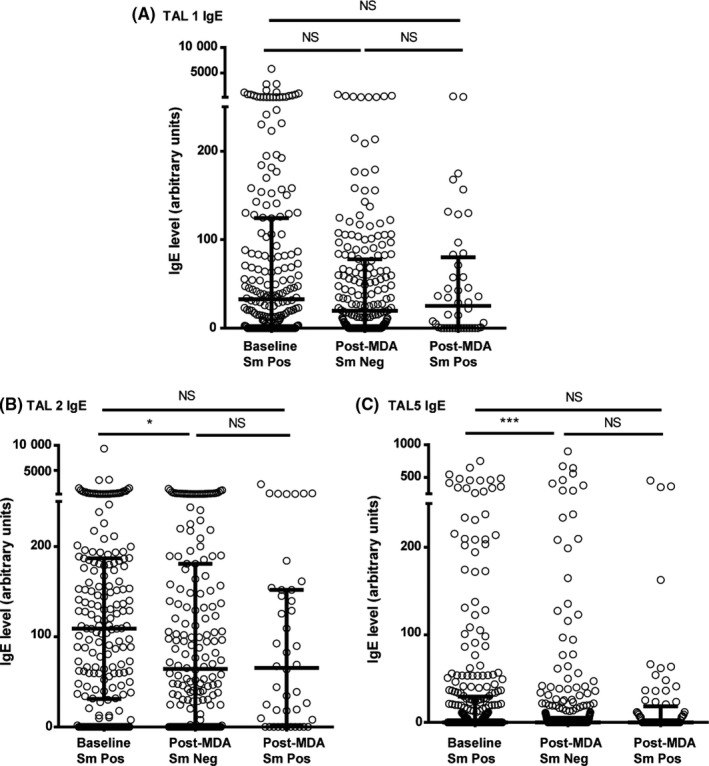
Anti TAL‐1 IgE l (A) and anti‐TAL‐2 IgE (B) and anti‐TAL‐5 IgE (C) levels at baseline for children who were all *Schistosoma mansoni* egg‐positive, and after 4 mass drug administrations (MDAs) (post‐MDA group) in children who were egg‐negative and those who were egg‐positive. Data are presented as dot plots, medians at centreline and whiskers at 25th and 75th percentile. Comparison was done by Kruskal‐Wallis followed by Dunn's multiple comparison test. *P < .05, **P < .01

Anti‐SEA IgE was significantly higher in the egg‐negative post‐MDA group than the baseline group. While median anti‐SWAP IgE was also higher in the post‐MDA group, this difference was not statistically significant, as shown in Figure 5. For the recombinant TAL antigens, median IgE levels were lower among egg‐negative children in the post‐MDA group, except for TAL‐1 for which IgE levels did not differ by group (Figure 6).

**Figure 7:**
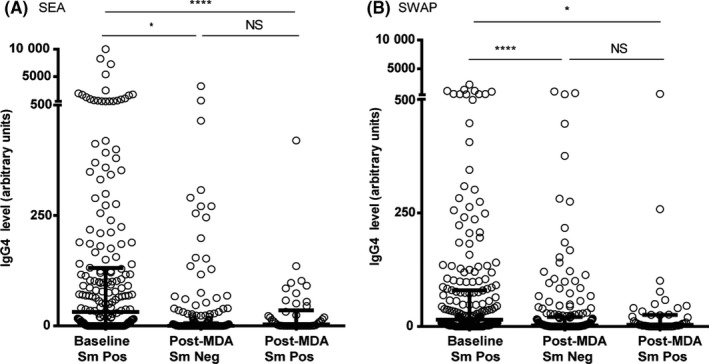
Anti‐(SEA) soluble egg antigen IgG4 levels (A) and soluble worm antigen preparation anti‐(SWAP) IgG4 levels (B) at baseline for children who were all *Schistosoma mansoni* egg‐positive, and after 4 mass drug administrations (MDAs) (post‐MDA group) in children who were egg‐negative and those who were egg‐positive. Data are presented as dot plots, medians at centreline and whiskers at 25th and 75th percentile. Comparison was done by Kruskal‐Wallis followed by Dunn's multiple comparison test. *P < .05, **^**^ *P* < .0001

**Figure 8:**
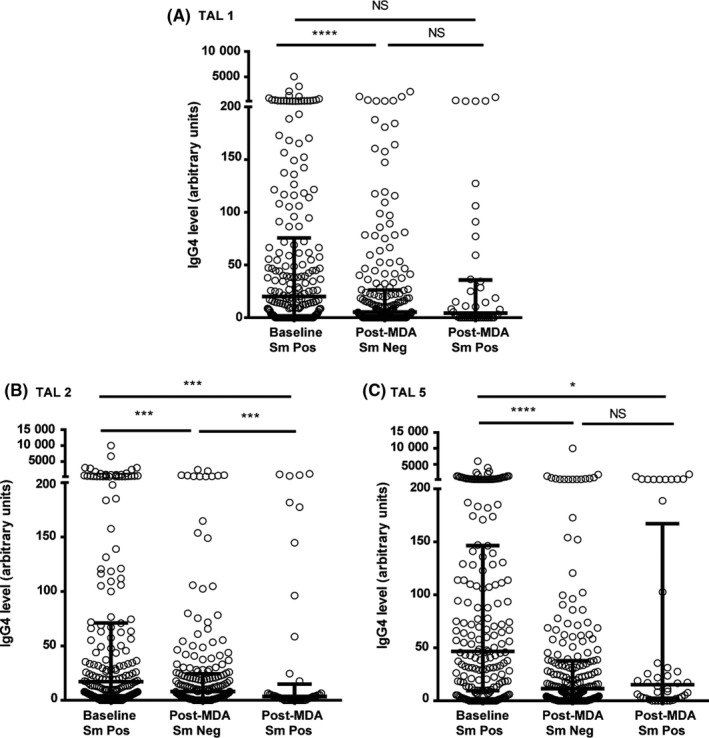
Anti TAL‐1 IgG4 l (A) and anti‐TAL‐2 IgG4 (B) and anti‐TAL‐5 IgG4 (C) levels at baseline for children who were all *Schistosoma mansoni* egg‐positive, and after 4 mass drug administrations (MDAs) (post‐MDA group) in children who were egg‐negative and those who were egg‐positive. Data are presented as dot plots, medians at centreline and whiskers at 25th and 75th percentile. Comparison was done by Kruskal‐Wallis followed by Dunn's multiple comparison test. *P < .05, **P < .005, *^**^ *P* < .0005 and **^**^ *P* < .0001

**Figure 9:**
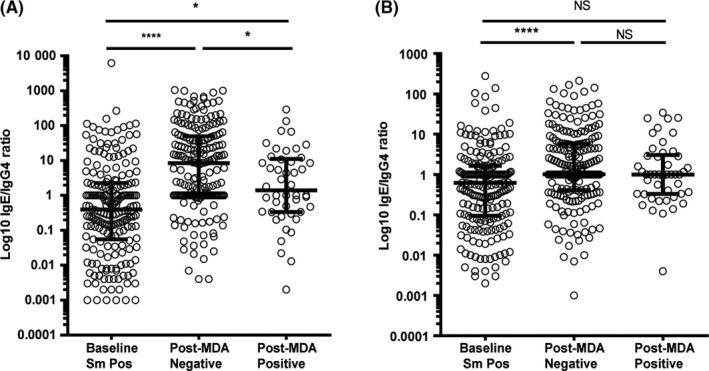
Anti‐(SEA) soluble egg antigen (A) and soluble worm antigen preparation anti‐(SWAP) (B) IgE/IgG4 ratios at baseline for children who were all *Schistosoma mansoni* egg‐positive, and after 4 mass drug administrations (MDAs) (post‐MDA group) in children who were egg‐negative and those who were egg‐positive. Data are presented as dot plots, medians at centreline, and whiskers at 25th and 75th percentile. Comparison was done by Kruskal‐Wallis followed by Dunn's multiple comparison test, with *P < .05 and **^**^ *P* < .0001

**Figure 10:**
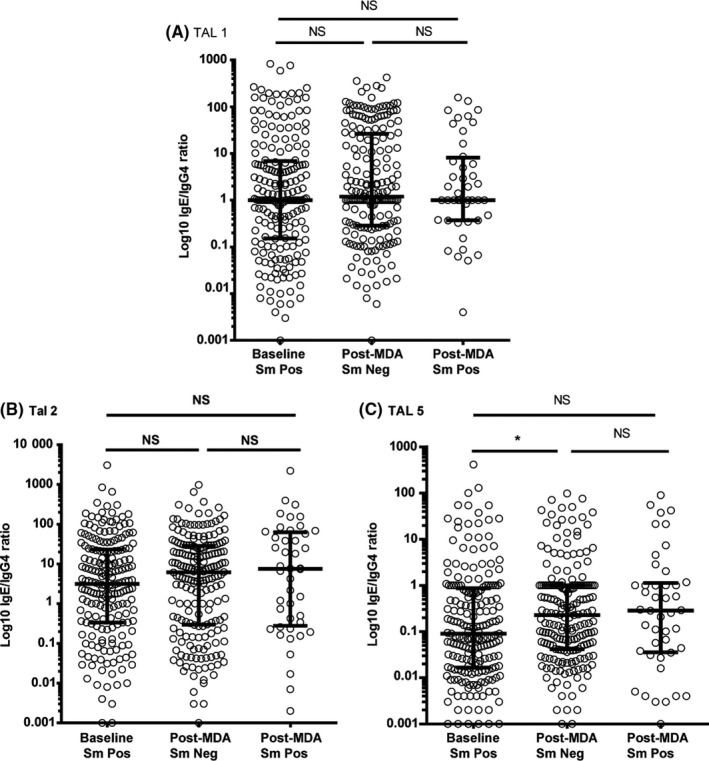
Anti‐TAL‐1 (A), anti‐TAL‐2 (B) and anti‐TAL‐5 (C) IgE/IgG4 ratios levels at baseline for children who were all *Schistosoma mansoni* egg‐positive, and after 4 mass drug administrations (MDAs) (post‐MDA group) in children who were egg‐negative and those who were egg‐positive. Data are presented as dot plots, medians at centreline and whiskers at 25th and 75th percentile. Comparison was done by Kruskal‐Wallis followed by Dunn's multiple comparison test. *P < .05

Both anti‐SEA and anti‐SWAP median IgG4 levels were significantly lower in the post‐MDA group (Figure 7). This was true whether children were egg‐positive or egg‐negative at the end of the study. Similarly, anti‐TAL‐1, anti‐TAL‐2 and anti‐TAL‐5 IgG4 levels were higher in the baseline group than in the post‐MDA group, more so, regardless of infection status for anti‐TAL 2 and anti‐TAL 5 (Figure 8). Median anti‐SEA IgE/IgG4 ratio in the post‐MDA group was significantly higher than in the baseline group, and children who were egg‐negative had higher anti‐SEA IgE/IgG4 ratio than those who were egg‐positive at that point. Uninfected children in the post‐MDA group also had significantly higher median anti‐SWAP IgE/IgG4 ratio than those in the baseline group (Figure 9). For the recombinant TAL antigens, the median IgE/IgG4 ratio of anti‐TAL‐5 antibodies was higher in the egg‐negative post‐MDA group than the baseline group, while these ratios for TAL‐1 and TAL‐2 were not different (Figure 10).

## 4 DISCUSSION AND CONCLUSION

Several studies have reported the beneficial effect of repeated treatment of schistosomiasis with PZQ following multiple reinfections on the development of partial resistance to reinfection by schistosomes in adults,^4^, ^5^, ^25^ as well as acquisition of antischistosome immunity following exposure to naturally dying worms.^1^, ^2^, ^25^ In this study, we investigated potential changes in immune responses in children attending schools where annual MDA with PZQ was provided for control of schistosomiasis. Immune responses of schistosome egg‐positive children before MDA were compared to children of the same age group and attending the same schools after 4 rounds of annual PZQ and albendazole treatment. Infection levels were markedly lower in the post‐MDA group, and we observed clear differences in several antischistosome immune responses between the baseline group and the group evaluated post‐MDA. The overall prevalence in these 5 schools at baseline, based on a single stool specimen, 2 slides each, was 44.7%.^24^ It is possible that the selection of only *S. mansoni* egg‐positive children at baseline may have introduced a degree of bias in regard to this group of children having a potentially higher degree of previous exposure than their uninfected counterparts. However, it is also likely in an area with this level of prevalence that many of the *S. mansoni* egg‐negative children not selected may have harboured light infections that were not detected by stool exam.

The higher than baseline levels of IFN‐γ production in response to SEA observed in egg‐negative children in the post‐MDA group suggests that SEA stimulation of IFN‐γ may be immunoregulated in untreated infections, and that multiple MDAs removed this regulation. Higher SEA‐stimulated IFN‐γ levels have mainly been reported in people with acute schistosomiasis, prior to the development of the regulation associated with chronic infection.^26^, ^27^, ^28^, ^29^ Wilson et al^30^ also reported a treatment‐associated increase in IFN‐γ, although this increase was only seen 1 year after treatment and was not sustained at 2 years post‐treatment.

Both SEA‐ and SWAP‐stimulated IL‐5 as well as SWAP‐stimulated IL‐13 levels were higher in children who were egg‐negative in the post‐ MDA group, suggesting that untreated infection at baseline was exerting a level of regulation of these Th2 cytokines. Clearing of infections with multiple annual MDAs may have removed this regulation, allowing for increased production of Th2‐related cytokines in immune‐sensitized but egg‐negative individuals. Wilson et al^30^ reported similar findings in their 2‐year follow‐up study in Kenya, attributing the increase in SEA‐stimulated Th2 cytokines after treatment to removal of downregulation associated with active infection, while they attributed increased worm antigen‐stimulated responses to antigenic exposure due to release of antigens upon treatment and possible increased immune memory to these antigens. The current study did not, however, find a difference in IL‐5 and IL‐13 production between infected and uninfected children in the post‐MDA group. Schistosome infection‐induced immunoregulation due to IL‐10 might also be inferred from the considerable spontaneous production of IL‐10 by unstimulated cells at baseline. In the post‐MDA group, there was much less spontaneous IL‐10 (*P* < .0001) production for both day 3 and day 5 cultures. The observation that in response to PHA, less IL‐10 was produced by whole‐blood cultures from the post‐MDA group compared to the baseline group, regardless of infection status (*P* < .0001), may indicate that PHA nonspecific mitogen stimulation of IL‐10 was not itself appreciably regulated by active infection. This was in contrast to SEA‐ and SWAP‐ stimulated production of IL‐10, which appeared to be downregulated in egg‐positive individuals, while still allowing strong specific SEA stimulation of IL‐10. This is in agreement with findings from a study of *Schistosoma haematobium* in children in Gabon that reported an increase, in some cases, of IL‐10 following multiple treatments.^31^ Joseph et al^8^ did not observe a difference in un‐stimulated levels of IL‐10 before and 7 weeks after treatment in a group comprised of children and adults although there was a significant increase in both SEA and SWAP‐stimulated IL‐10 at this time‐point.

Parasite‐specific IgE is one of the most consistent immune responses associated with protection against reinfection by schistosomes.^32^ Antiworm IgE increases after treatment^3^ and repeated treatment following reinfections appear to augment protective responses, allowing increasingly longer durations to reinfection.^4^, ^5^ We found higher anti‐SEA IgE among children following multiple MDAs compared to egg‐positive children at baseline. This may be indicative of infection‐driven regulation of anti‐SEA IgE at baseline. Post‐treatment increases in anti‐SEA IgE have also been reported in children treated for *S. haematobium*.^11^ Anti‐SWAP IgE was only marginally higher than baseline in the post‐MDA group in this study, while no or lower levels of IgE against the adult worm‐associated recombinant antigens TAL‐1 and TAL‐5 were observed in the post‐MDA group. Anti‐TAL‐2 IgE was also lower in the post‐MDA group. TAL‐2 is an antigen that is expressed throughout the life cycle of the schistosome.^21^ Previous studies have reported a post‐treatment increase in anti‐SWAP and anti‐TAL‐1 and TAL‐5 IgE in association with resistance to infection.^25^, ^32^, ^33^, ^34^ In the current study, the children at the final year (5th year from baseline) of study may not have had multiple reinfections even though their schools received 4 MDAs. It may take multiple treatments and reinfections to augment protective responses.^4^ Data also support an effect of age on development of protective responses,^35^, ^36^, ^37^ which may have been limited in the age group in our study. It should be noted that possible discrepancies between the current results and those previously published, such as IgE to SWAP, TAL1 and TAL5 following treatment^25^, ^32^, ^33^, ^34^ could well be due to the current study focusing on a time frame of years including multiple treatments as opposed to weeks or months following a single treatment.

IgG4 is another important antibody isotype response to schistosome antigens. IgG4 levels against both crude and recombinant antigens were lower than baseline in the post‐treatment group, especially in the uninfected children. This is consistent with what has been reported elsewhere.^38^, ^39^ Concurrent with post‐treatment increases in protective antischistosome IgE, a post‐treatment reduction in antischistosome IgG4 is also associated with protection against reinfection.^7^ Others have suggested that it is the balance between IgE and IgG4 that is important in resistance or susceptibility to schistosome infection.^40^, ^41^ Consistent with this hypothesis, we found higher anti‐SEA, anti‐SWAP and anti‐TAL‐5 IgE/IgG4 ratios in the post‐MDA group especially for individuals who were egg‐negative at the final time‐point.

Findings from this study demonstrate that multiple annual primary and secondary school‐based MDA with PZQ, as recommended by the WHO, likely alters schistosome‐specific immunological responses of the children in those schools. Most notable is the apparent removal of schistosome‐specific immunoregulation associated with the baseline infections. Our study was specifically designed to evaluate potential immunologic changes in populations of children in schools receiving multiple annual rounds of MDA. It was not designed to longitudinally follow‐up individual children and cannot determine if a child experienced multiple reinfections during the 5‐year study period following each of the 4 annual MDAs their schools received. However, our results are consistent with the hypothesis that in addition to reducing prevalence and intensity of infection, multiple school‐based MDAs effect changes in the immune responses of most of the children in those schools, potentially increasing their resistance to reinfection upon subsequent exposures as well as decreasing their schistosome worm burden and egg output to the environment.^42^

## DISCLAIMER

This study is published with the permission of the Director of the Kenya Medical Research Institute.

The findings and conclusions in this report are those of the authors and do not necessarily represent the views of the Centers for Disease Control and Prevention.

## DISCLOSURES

None.
