# Supplementary figures and images for: Effect of four rounds of annual school‐wide mass praziquantel treatment for schistosoma mansoni control on schistosome‐specific immune responses
Source: Parasite Immunol. 2018 Apr 29;40(6):e12530. doi: 10.1111/pim.12530 (PMC6001474; doi:10.1111/pim.12530)

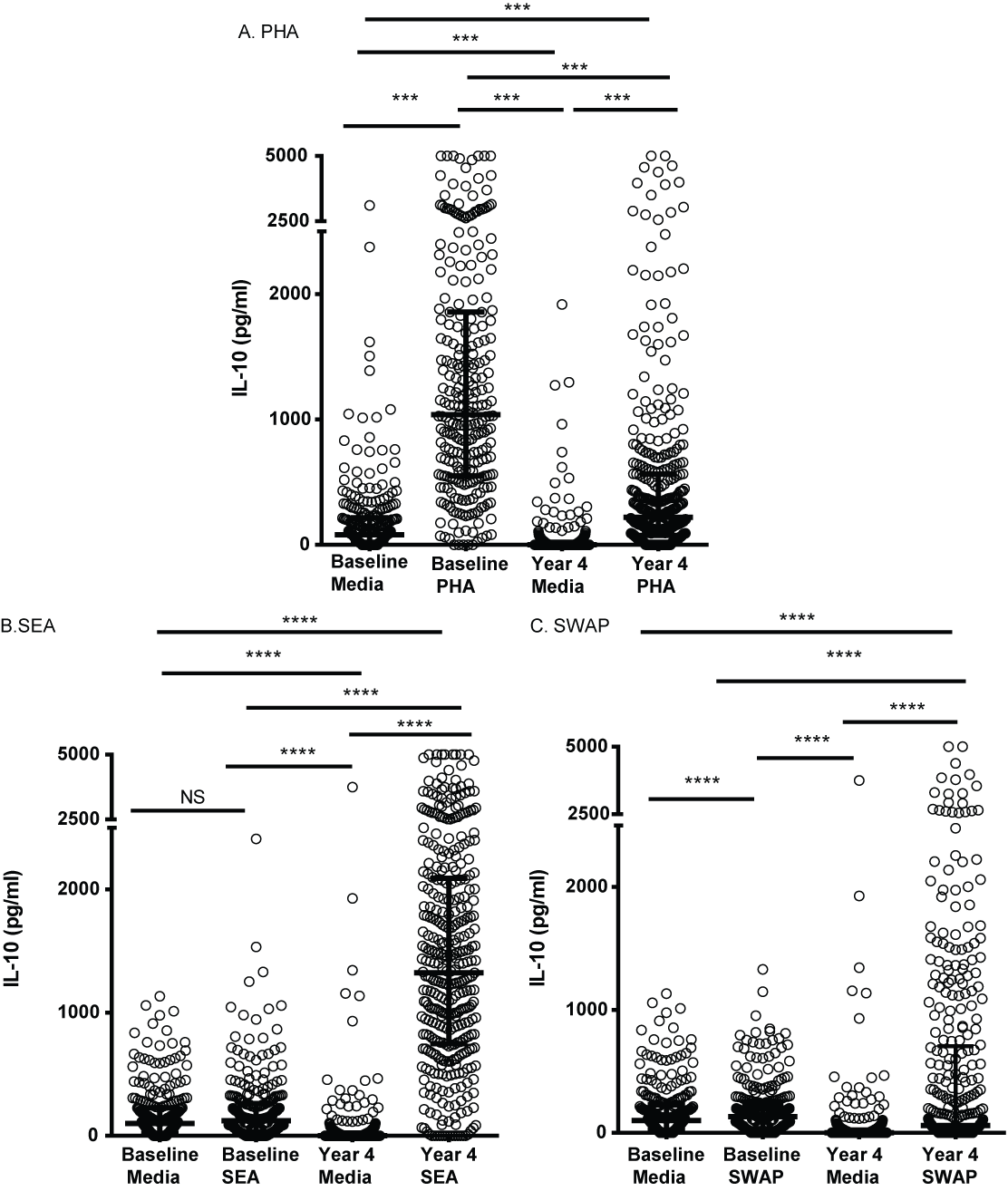

Supplement: Supplementary file 1 [file PIM-40-na-s001.tif]
